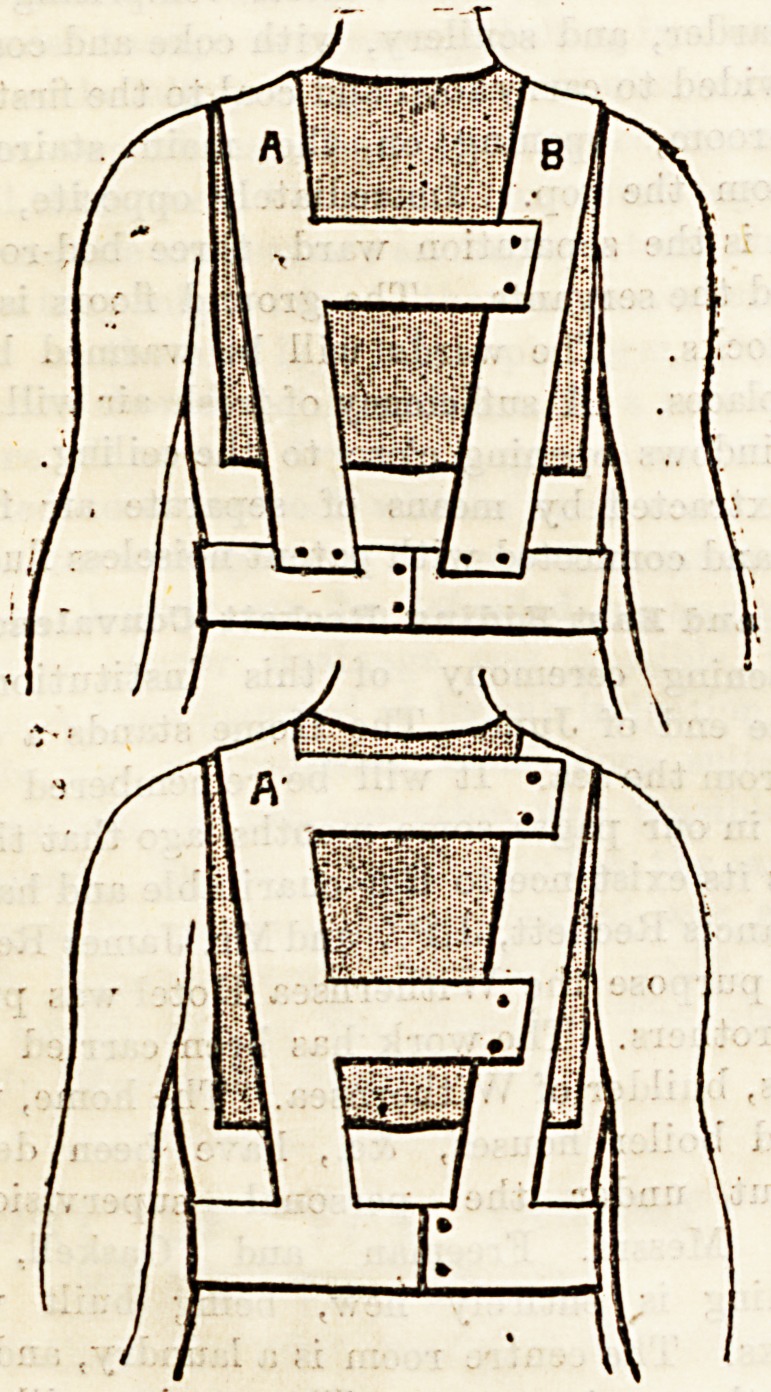# Practical Experience in Poultice Making

**Published:** 1893-09-02

**Authors:** 


### EDITOR'S LETTER-BOX.

[Our correspondents are reminded that proxility is a great bar to publication, and that brevity of style and conciseness of statement greatly facilitate early insertion.]

### PRACTICAL EXPERIENCE IN POULTICE MAKING.

"AN UNPROFESSIONAL READER" writes: I think it may interest some of your readers to hear of a little invention for retaining poultices in place that I put into practice last year when nursing my husband abroad, through an acute attack of bronchitis and congestion of both lungs, consequent upon influenza.

I was ordered to keep on poultices day and night, back and front. But the question was how to carry out the orders. My only apparatus was a small Etna and two diminutive saucepans, some four inches deep, out of which I had already turned out, as I thought, fair sized ones. As I gazed in dispair from these small receptacles to the vast expanse of masculine back and chest that was to be covered my heart sank within me. However, by dint of relays of saucepans with boiling water, plenty of linseed meal and oil-silk, that difficulty soon bid fair to be overcome, but the "crux" was how to keep them on with a patient who utterly refused to be coddled and wanted to sit up in bed.

Suddenly an idea occurred to me, and hastily swathing my patient up in the only available garment, I seized some stout calico, and tearing it into strips, which I folded in half, I set to work with needle and thread to make what, for want of a better name, we will call a pair of poultice braces, for it was the sight of my husband's red braces, and an insane desire in some way to strap the poultice on with them, that gave me the notion.

"How did you get him into them?" perhaps asks some inquiring mind on contemplating the annexed diagram—which otherwise will speak for itself. Why, button the band round his waist, throw the two uprights over his shoulders, buttoning them to the buttons behind, fastening and unfastening the cross-pieces only when you want to slip the poultice in and out. A safety pin at A and B keep the poultices in place, and two are better than one, as they counterbalance each other in weight.

Well, I can only say that we used those "poultice-braces" day and night, sitting up or lying down, with a piece of oil-silk slipped in over them, and not a poultice shifted or got cold before its appointed time, while one was slipped out and another in with the least possible trouble and inconvenience.

I waive all copyrights in favour of the public, and only add, "Try them."

If a poultice is desired under the arm, two counterbalancing strips from side to side hold it in place equally well.

## Figures and Tables

**Figure f1:**